# Identification and Evaluation of Cancer Stem Cells in Oral Squamous Cell Carcinoma and Oral Epithelial Dysplasia Using NANOG: An Immunohistochemical Study

**DOI:** 10.7759/cureus.55111

**Published:** 2024-02-27

**Authors:** Arya I, Varun B Raghavan Pillai, Anna P. Joseph, Pratibha Ramani, Jayanthi P, Karthikeyan Ramalingam

**Affiliations:** 1 Oral and Maxillofacial Pathology, PMS College of Dental Science and Research, Trivandrum, IND; 2 Oral Pathology and Microbiology, Saveetha Dental College and Hospitals, Saveetha Institute of Medical and Technical Sciences, Saveetha University, Chennai, IND; 3 Oral and Maxillofacial Pathology, Azeezia College of Dental Sciences and Research, Kollam, IND

**Keywords:** expression, progression, potentially malignant, prognosis, marker, oral leukoplakia, immunohistochemistry, oscc, oral epithelial dysplasias, cancer stem cells

## Abstract

Background: Squamous cell carcinoma of the oral cavity may show precursor lesions, termed as potentially malignant disorders, of which leukoplakia is the most frequent one. Oral leukoplakia is a clinical diagnosis for which the histological diagnosis may be either hyperplasia or oral epithelial dysplasia (OED) and sometimes even oral squamous cell carcinoma (OSCC). Cancer stem cells (CSCs), identified in various tumors, are a specific group of cells that exhibit the properties of self-renewal and differentiation. Among the various biomarkers that identify CSCs, the transcription factor NANOG is considered to be a significant one.

Aim: In this study, we intend to identify and compare the immunohistochemical expression of NANOG in OSCC, OED, and normal oral mucosa.

Methodology: Tissue blocks of OSCC (n=28), OED (n=28), and normal oral mucosa (n=28) were used in this study. Specimens were immunohistochemically analyzed for NANOG expression. The results were statistically analyzed using one-way ANOVA, Games-Howell post hoc, and Student t-test. Statistical Product and Service Solutions (SPSS, version 21; IBM SPSS Statistics for Windows, Armonk, NY) software was used for performing the statistical analysis, and the level of significance was set as 0.05.

Observations: NANOG expression was higher in OSCC when compared to oral dysplasias and normal oral mucosa, in decreasing order. A significantly higher histo-score and labeling index score were observed in OSCC and oral dysplasias compared to normal oral mucosa (p=<0.001).

Conclusion: The expression levels of NANOG were positively correlated with disease progression in OSCC, implicating that NANOG can be used as a surrogate marker of oral oncogenesis and prognosis. Therefore, decoding the molecular mechanisms of NANOG regulation in the progression of cancer helps in developing new therapeutic strategies for oral cancer.

## Introduction

Oral squamous cell carcinoma (OSCC) forms the major type of cancer in the head and neck region and poses a significant threat to the well-being of the patient [1,2]. The cumulative incidence of oral cancer is four per 100,000 persons with approximately 350,000 cases diagnosed each year. Different nations around the globe exhibit varied prevalence of OSCC and in developing countries, such as India, around 30% of oral cancer cases are diagnosed every year [3,4]. A staggering 22.9% of cancer deaths in India are attributed to OSCC [5,6].

In about 15%-45% of the cases, oral cancer might show visible precursor lesions, of which oral leukoplakia (OL) is the most frequently occurring lesion of the mouth. Oral leukoplakia is predominantly a non-scrapable white patch clinically diagnosed after excluding other possible causes of white lesions in the mouth [7]. Histopathologically, OL may exhibit features of either hyperplasia or oral epithelial dysplasia (OED) and sometimes even frank carcinoma. Even though OL has multifactorial etiologies, tobacco is considered to be the most common causative agent. The prevalence of leukoplakia ranges between 1.5% and 4% throughout the world, whereas, in India, this range is wider with an estimated value of 0.2%-4.8% [8,9].

Cancer stem cells (CSCs) have been identified in tumors and exhibit self-renewal and differentiation properties. It has been demonstrated that, when these cells are transplanted into an animal, they have the capacity for tumor formation [10]. In the year 1994, John Dick and his co-workers first identified cancer stem cells in neoplasms of hematolymphoid origin [11]. In later years, these cells were found in various primary malignant tumors and in OSCC cell lines. The presence of cancer stem cells was associated with poor prognosis as the result of their involvement in lymph node metastasis and local recurrence. CSCs in OSCC express proteins such as NANOG, OCT4, and SOX2, which regulate embryonic stem cells [12,13].

NANOG is a stem cell transcription factor involved in human embryogenesis, cell fate determination, proliferation, and apoptosis. The expression of NANOG is very low or absent after birth and remains so throughout the lifespan. However, NANOG expression is detectable in a small proportion of cancer cells that show stem cell-like properties. Higher expression of NANOG has been observed in various solid tumors of the breast, colon, lung, liver, and cervix. Elevated values of NANOG especially in cancer cells were described by previous research, which means that the NANOG gene is a critical member of cancer stem cells [14]. This study was done to evaluate the cancer stem cells in OSCC and OED using immunoexpression of NANOG.

## Materials and methods

Sample size calculation was performed using the reported data [14,15] due to a lack of relevant literature. The tissue sections of oral leukoplakia with OED (n=28), OSCC (n=28), and normal oral mucosa (n=28) were taken from the archival blocks stored in the Department of Oral Pathology Department at PMS Dental College, Trivandrum, India. 

Ethical clearance was obtained from the institutional ethical committee before conducting this study (PMS/IEC/2020-21/01). Clinically recorded cases of leukoplakia, which are histopathologically graded as epithelial dysplasia, and histopathologically confirmed cases of OSCC were included in the study. Biopsy specimens from both genders falling under the age group of 20-70 years were included. The tissue sections without epithelium were not considered for this particular study. Tissue specimens of normal oral mucosa harvested during surgical removal of impacted third molars constituted the control group. Those individuals with any systemic illness or any known inflammatory conditions about the third molar were excluded.

Four micron-thick tissue sections were made and transferred onto the aminopropyltriethoxysilan (APES)-coated slide. Seminoma was kept as positive immunohistochemistry (IHC) control. Deparaffinization was done using xylene for 10 minutes, followed by rehydration, with the help of descending grades of alcohol (100%, 90%, and 70%). The slides were then placed in a jar containing Tris EDTA buffer (pH-9), and antigen retrieval was performed in a 5 L pressure cooker at 100°C. After blocking the endogenous peroxidase, the tissue sections were incubated overnight with rabbit anti-NANOG monoclonal antibody (D73G4, Scientific solutions, 1:800 (diluent-signal stain antibody diluent #8112)). After washing with an immuno-wash buffer, the slides were incubated with polyExcel HRP secondary antibody for 30 minutes and with diaminobenzidine (DAB) for 30 minutes. Finally, the sections were counterstained with hematoxylin and observed under light microscopy.

Two independent pathologists performed the analysis of NANOG expression through light microscopy at a magnification of 40x and evaluated the number of positive and negative cells. The manual cell counting was done in five consecutive high-power fields for each slide, and cell counts were averaged. The areas with strong NANOG expression were selected for cell counting. In OSCC, NANOG-positive cells were evaluated mainly in the tumor-invading front, whereas in OED and normal mucosa, positive cells were evaluated within the epithelium. Testicular seminoma was taken as the positive control to compare the expressions of staining intensities.

For analysis of the staining results in OSCC, OED, and normal mucosa, a semi-quantitative approach using histo-score (H-score) was used [15]. The NANOG expression was assessed based on the nuclear and cytoplasmic staining of tumor cells, and the percentage of tumor cells was counted. The intensity score was considered negative for cells that did not show any staining (score 0), weak staining less than that of positive controls (score 1), moderate staining equivalent to positive control (score 2), and intense staining stronger than positive control (score 3). The following formula was used to assign the percentage of cells at each staining intensity level: {[1 × % cells 1+] + [2 × % cells 2+] + [3 × %cells 3+]}. The percentage of positive cells was calculated as the labeling index (LI), which was derived by adding up the total number of NANOG-expressing tumor cells and dividing that value by the total number of tumor cells [16].

Statistical Product and Service Solutions (SPSS, version 21; IBM SPSS Statistics for Windows, Armonk, NY) software was used to perform all the statistical analyses. Comparison between the groups was done with one-way ANOVA and Games-Howell post hoc analysis. Comparison between the various grades of OED and OSCC was done with Student's t-test, and a p-value less than 0.05 was considered to be statistically significant.

## Results

Retrospective analysis of our archival records revealed that, among the 28 samples of OSCC, 20 were males, eight were females, and they belonged to the age group between 40 and 85 years. Among the 28 samples of OED, 19 were males, nine were females, and they belonged to the age group between 20 and 90 years. Among the 28 samples of normal mucosa, 18 were males, 10 were females, and they belonged to the age group between 20 and 50 years. Chewing tobacco was the most prevalent deleterious habit (87%) noted in both OSCC and OED groups. Smoking tobacco was noted in the remaining 13% of both OSCC and OED groups.

The biopsy specimens of OSCC and OED were taken from different anatomic sites within the oral cavity. Out of the 28 samples of OSCC, nine were located on the buccal mucosa, 12 on the tongue, four on the alveolar mucosa, and three on the gingiva. Out of the 28 samples of OED, 10 were located on the buccal mucosa, 11 on the tongue, five on the alveolar mucosa, and two on the gingiva.

**Figure 1:**
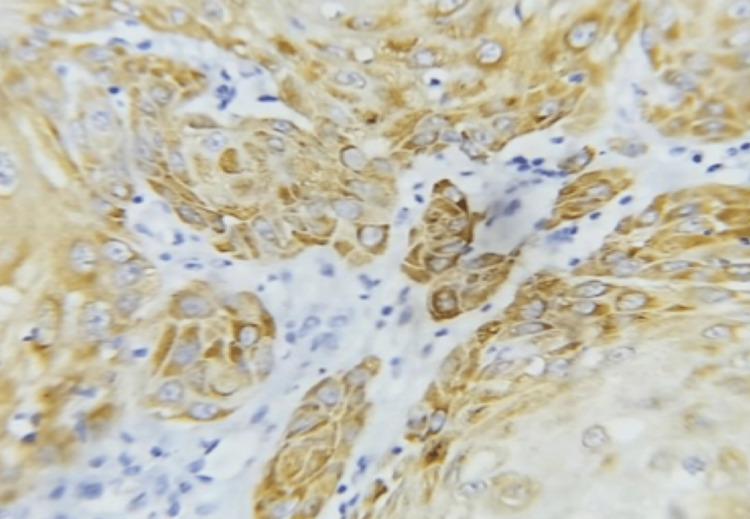
Photomicrograph of diffuse NANOG immunohistochemical expression in squamous cell carcinoma (IHC, 40x)

**Figure 2:**
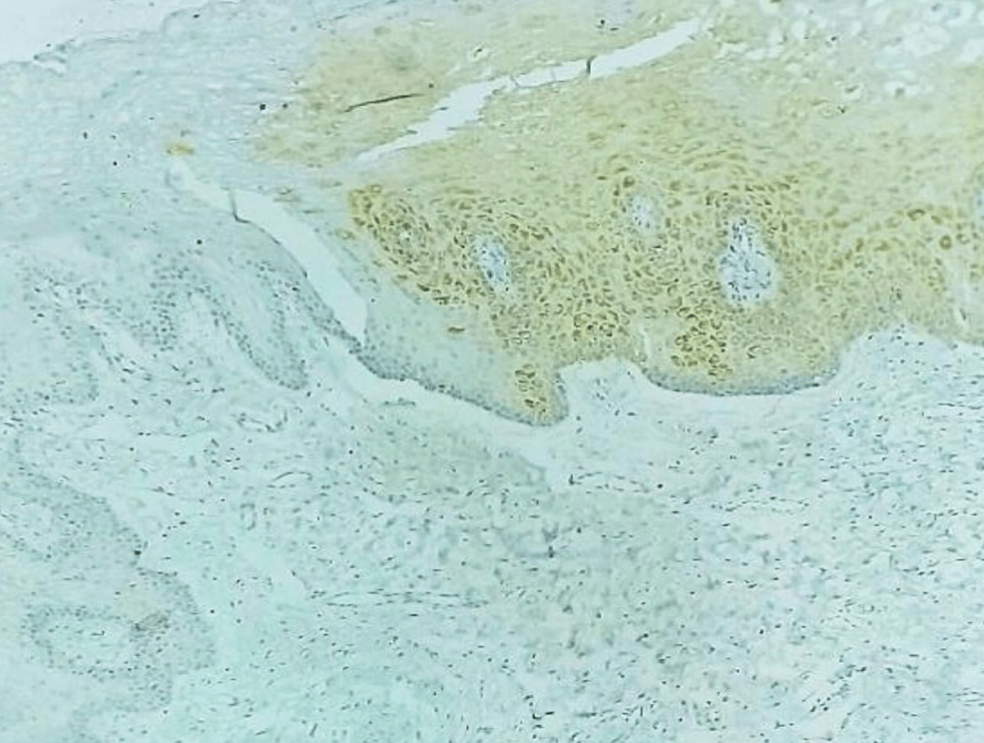
Photomicrograph of focal NANOG immunohistochemical expression in epithelial dysplasia (IHC, 10x)

**Figure 3:**
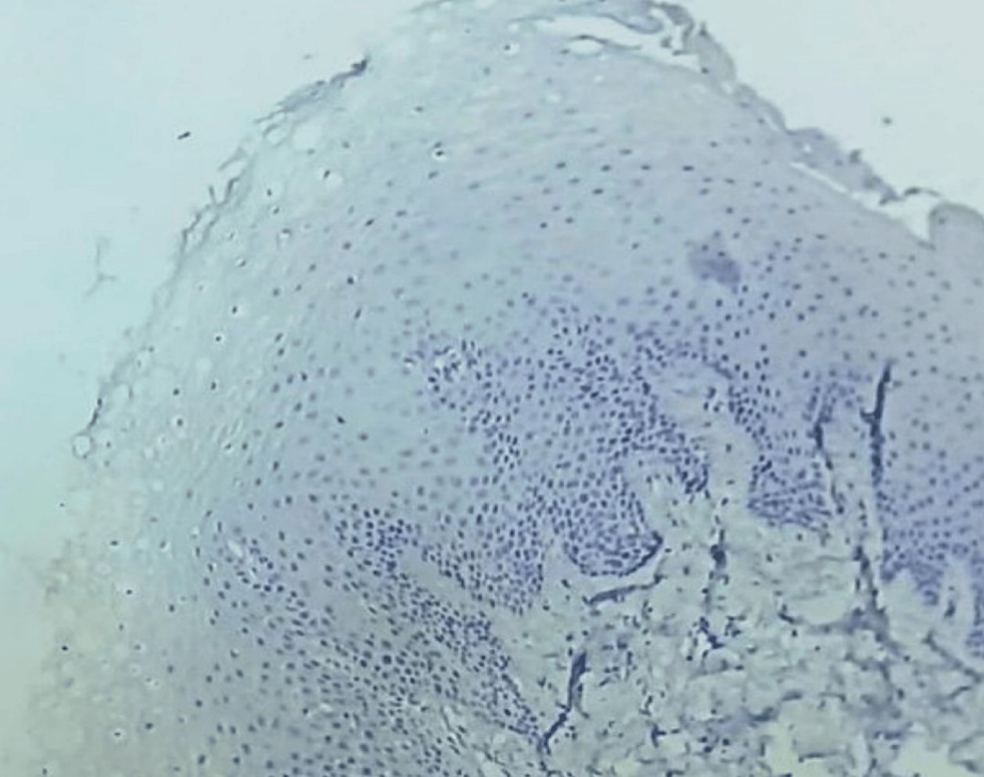
Photomicrograph showing low NANOG immunohistochemical expression in normal epithelium (IHC, 20x)

**Figure 4:**
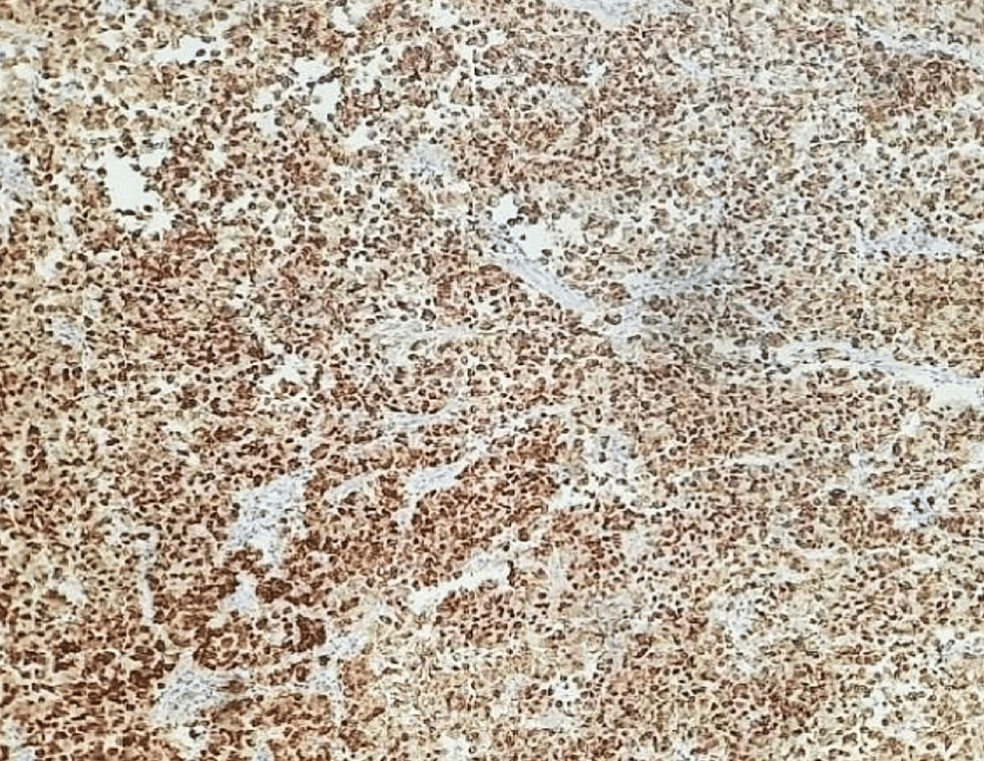
Photomicrograph of NANOG immunohistochemical expression in the positive control of testicular seminoma (IHC, 10x)

All sampled categories, such as OSCC, OED, and normal oral mucosa, were compared for NANOG expression using the immunohistochemical scoring analysis, H-score, and labeling index (LI). Photomicrographs of the study groups and control show the NANOG expression (Figures 1-4).

**Figure 5:**
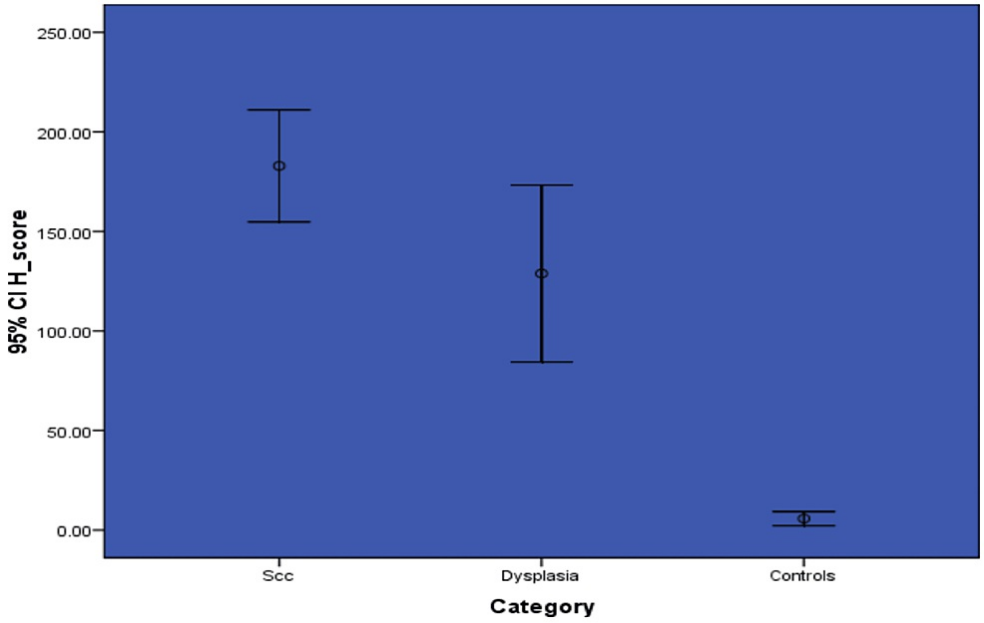
Graph showing the comparison of H-score of NANOG among the study groups SCC - Squamous Cell Carcinoma

When comparing the mean value of the H-score and LI score between the three groups, the mean H-scores of OSCC, OED, and normal mucosa were 182, 138, and 5.7, respectively (Figure 5). A significantly higher H-score was observed in OSCC when comparing all three study groups (p=<0.001).

**Figure 6:**
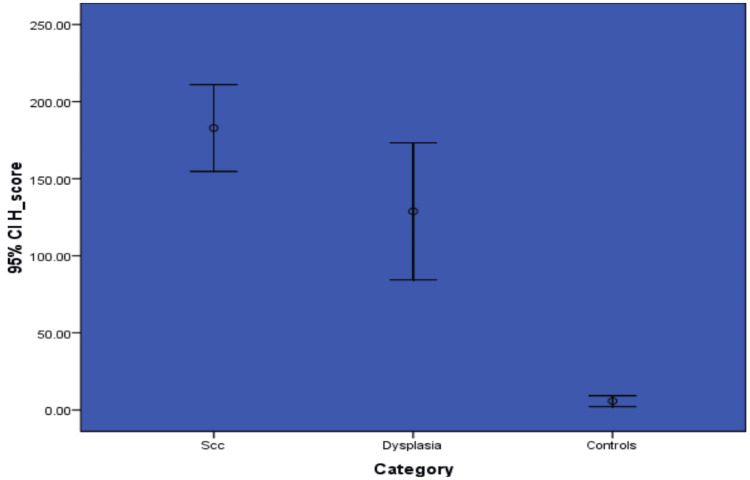
Graph showing the comparison of the LI scores of NANOG among the study groups SCC - Squamous Cell Carcinoma

The mean LI scores of OSCC, OED, and normal oral mucosa were 74, 48, and 2.5, respectively. The LI was significantly higher in OSCC than in OED and normal mucosa (p=<0.001) (Figure 6).

When comparing the H-score and LI scores among well-differentiated (n=12) and moderately differentiated (n=16) OSCCs, the following results were obtained. In OSCC, the expression was mainly noted in the invasive tumor front and within the tumor islands in the connective tissue. In OED, the expression of NANOG was seen throughout the epithelium, whereas in normal mucosa, it was restricted to the basal and para-basal layers of the epithelium. The mean H-score and LI were higher in moderately differentiated tumors, as compared to well-differentiated tumors. However, these differences were not statistically significant (Table 1).

**Table 1:** Comparison of NANOG expression using the H score and labeling index among different grades of OSCC with Student's t-test OSCC - Oral Squamous Cell Carcinoma

Student's t-test			
Category	Groups	Mean (SD)	p-value
H-score	Well-differentiated OSCC	173.2 (80)	0.514
	Moderately differentiated OSCC	190.8 (67.8)	
Labeling Index (LI)	Well-differentiated OSCC	74.63 (24.6)	0.889
	Moderately differentiated OSCC	73.55 (16.2)	

The mean value of the H-score was higher in high-risk OED (n=11) than in low-risk OED (n=17), and the difference was statistically significant. The statistical analysis between OSCC and OED was done using the Games-Howell post-hoc test. The H-score and LI score of NANOG were significantly higher in OSCC compared to those in OED (p=0.02). Though the mean value of the LI score was higher in high-risk OED than in low-risk OED, a statistical significance could not be obtained (p=0.071) (Table 2).

**Table 2:** Comparison of NANOG expression using the H score and labeling index among different grades of dysplasia by Student's t-test * - statistically significant value: p<0.05

Student's t-test			
Category	Groups	Mean (SD)	p-value
H-score	Low-risk dysplasia	88.08 (36)	0.02*
	High-risk dysplasia	188 (63.2)	
Labeling Index (LI)	Low-risk dysplasia	35.15 (14.3)	0.07
	High-risk dysplasia	60.8 (25)	

## Discussion

Squamous cell carcinoma of the oral cavity forms the predominant group of malignant neoplasms in the mouth [17]. Most OSCCs arise from precursor lesions, known collectively as potentially oral malignant disorders, of which OL is the most common one with a malignant transformation rate of around 1.1%-40.8% [18]. Early identification and prompt diagnosis of leukoplakia might be essential to control their transformation into malignancy. Microscopic evaluation and grading of dysplasia is still considered to be the best practical method for predicting malignant conversion. However, the major disadvantage of the currently used grading system is the intraobserver and inter-observer variability. Therefore, continuous search and research to identify accurate biomarkers that can predict the malignant transformation of oral leukoplakia are necessary.

Cancer stem cells are involved not only in the initiation and progression of tumors but are associated with lymph node metastasis and recurrence. Studies have shown that the transcription factor NANOG is a key regulator of CSC properties in carcinomas of the head and neck region. NANOG activates various signaling pathways to promote angiogenesis and also blocks apoptosis. In addition, NANOG was shown to reduce the expression of E-cadherin levels, which might lead to metastasis [19].

Research studies in various cancers have shown over-expression of NANOG. Meng et al. studied the expression of NANOG in colorectal carcinoma and showed that a low survival rate is directly correlated with higher levels of NANOG [20]. Nagata et al. found that NANOG expression levels were favorable prognostic factors for triple-negative breast cancer patients [21]. The combined expression of NANOG with other CSC markers such as OCT-4, and SOX-2, and proliferation markers, such as KI-67 and PCNA, were better predictors of relapse in patients with gastric cancer, as demonstrated by Li et al. [22]. The positive expression of NANOG in serous carcinoma of the ovary was correlated with poor prognosis by Lee et al. [23]. Zhao et al. linked the high levels of NANOG protein to high-grade tumors and metastatic lymph nodes in intestinal-type gastric cancer [24]. All these studies have shown that NANOG may be considered a potential prognostic marker in most primary tumors. However, only a few studies are found in the literature that compare NANOG expression in OSCC and epithelial dysplasia. Hence, in this present study, we have addressed this lacuna.

In our study, NANOG expression was noticed in 71.4% of dysplasia cases, and all these samples showed cytoplasmic staining of cells. When comparing the NANOG expression in different grades of OED, the expression was found to be higher in high-risk dysplasias than in low-risk dysplasias. This is consistent with the previous studies of de Vicente et al., where increased NANOG expression was observed in higher grades of dysplasia. In their study, 55 cases of OED were analyzed for immunohistochemical expression of NANOG protein, and the results showed that 3.6% and 16.4% of cases exhibited nuclear and cytoplasmic staining, respectively. Additionally, after a mean follow-up of 185 months, patients who harbored NANOG-positive cells showed significantly higher chances for malignant progression to oral cancer [15].

In the current study, NANOG protein expression was positive in 92.8% (n=26/28) of OSCC cases, all of which were primarily observed in the cytoplasm of tumor cells, whereas in germ cell tumors, a nuclear staining pattern was seen. Our results are similar to the study conducted by Grubelnik et al. who assessed both the protein and mRNA expressions of NANOG in 30 samples of OSCC and found that 27 cases exhibited positive staining mainly in the cytoplasm of tumor cells [14].

Our study also demonstrates that the expression of NANOG is increased in higher grades of OSCC and high-risk OED similar to previous studies [25-28]. The reason for the two different staining patterns remains unclear. However, Gu et al. conducted a study on cervical cancer and showed that cellular localization of NANOG was related to the stage of the tumor and the type of cell involved. They suggested that the stromal cytoplasmic NANOG staining was associated with the progression of cervical cancer [28]. Therefore, our study uncovers the potential application of NANOG expression as an early surrogate marker for predicting malignant conversion in leukoplakia and a potential prognostic indicator in OSCC.

The limitations of our study were the retrospective nature of samples and a single-center evaluation of NANOG expression. In our study, the NANOG protein level was higher in OSCC compared to OED and normal mucosa using IHC. Even though there was an overall increase in the immunoexpression of NANOG in OSCC, few specimens showed lower expressions when compared to OED and normal mucosa. We also did not specifically analyze other histopathological parameters due to differences in the composition of study groups. The number of samples within each grade of OSCC was less, and, hence, a statistically significant difference in NANOG expression between moderately differentiated and well-differentiated OSCC could not be achieved. Further, our study samples did not include poorly differentiated OSCC, and the expression of NANOG in high-grade tumors could not be ascertained in this study. The increased expression of NANOG in high-risk dysplasias compared to low-risk dysplasias uncovers the potential of NANOG as a surrogate marker for malignant transformation into OSCC. However, longitudinal follow-up studies on a larger sample size would help us assess the exact role of NANOG in the malignant transformation and prognosis of OSCC and OED. Multiple institutions across the globe should be involved to assess the clinico-pathological correlation of NANOG expression with clinical presentation, regional invasion, distant metastasis, recurrence, disease-free survival, and mortality rate. NANOG expression in various potentially malignant disorders, along with continuous monitoring, will shed light on its prognostic ability as a biomarker. In the future, longitudinal follow-up studies on a larger sample size would help us assess the exact role of NANOG in the malignant transformation and prognosis of OSCC and OED.

## Conclusions

This study identifies the presence of cancer stem cells using immunohistochemical expression of NANOG in OSCC, compared with OED and normal mucosa. In conclusion, NANOG expression levels seem to be positively correlated with disease progression in OSCC, implicating that NANOG can be used as a biomarker of oral oncogenesis and prognosis. NANOG can also be used as a biomarker to predict the progression of the disease from dysplasia to OSCC. Therefore, decoding the molecular mechanisms of NANOG regulation can help in developing new therapeutic strategies for oral cancer.
